# *N*-Acetylcysteine Reduces Markers of Differentiation in 3T3-L1 Adipocytes

**DOI:** 10.3390/ijms12106936

**Published:** 2011-10-19

**Authors:** Pablo Calzadilla, Daiana Sapochnik, Soledad Cosentino, Virginia Diz, Lelia Dicelio, Juan Carlos Calvo, Liliana N. Guerra

**Affiliations:** 1 Departamento de Química Biológica, Facultad de Ciencias Exactas y Naturales, Universidad de Buenos Aires, Intendente Güiraldes 2160, Pabellón 2, Buenos Aires 1428, Argentina; E-Mails: pablo_calza@hotmail.com (P.C.); daianasapochnik@hotmail.com (D.S.); solecosen@hotmail.com (S.C.); juancalvo@ibyme.conicet.gov.ar (J.C.C.); 2 Departamento de Química Inorgánica y Analítica, Facultad de Ciencias Exactas y Naturales, Universidad de Buenos Aires, Intendente Güiraldes 2160, Pabellón 2, Buenos Aires 1428, Argentina; E-Mails: virginiadiz@yahoo.com.ar (V.D.); led@qi.fcen.uba.ar (L.D.); 3 IBYME-CONICET, Vuelta de Obligado 2490, Buenos Aires 1428, Argentina

**Keywords:** NAC, triglyceride, adipocyte differentiation

## Abstract

Oxidative stress plays a critical role in the pathogenesis of diabetes, hypertension and atherosclerosis. Some authors reported that fat accumulation correlates to systemic oxidative stress in humans and mice, but the relationship of lipid production and oxidative metabolism is still unclear. In our laboratory we used 3T3-L1 preadipocytes, which are able to differentiate into mature adipocytes and accumulate lipids, as obesity model. We showed that intracellular reactive oxygen species (ROS) and antioxidant enzymes superoxide dismutase (SOD) and glutathione peroxidase (GPx) activities increased in parallel with fat accumulation. Meanwhile *N*-acetylcysteine (NAC), a well known antioxidant and Glutathione (GSH) precursor, inhibited ROS levels as well as fat accumulation in a concentration-dependent manner. NAC also inhibited both adipogenic transcription factors CCAAT/enhancer binding protein beta (C/EBP β) and peroxisomal proliferator activated receptor gamma (PPAR γ) expression; we suggested that intracellular GSH content could be responsible for these effects.

## 1. Introduction

Obesity is a condition of pandemic proportions, against which no effective pharmacological treatment has yet been found. This condition is characterized mainly by the excessive accumulation of triacylglycerol (Tg) in white adipose tissue, which result in larger adipocytes, inflammatory responses and changes in the adipokines secreted. The murine 3T3-L1 fibroblastic cell line can be induced to differentiate into mature adipocytes in cell culture using dexamethasone and insulin [1], and it has been used as a model system to study the mechanisms involved in the adipogenic process. Mac Dougald *et al.* demonstrated that adipocyte differentiation program is regulated by transcriptional activator such as CCAAT/enhancer binding protein beta (C/EBP β) and peroxisomal proliferator activated receptor gamma (PPAR γ), which are able to coordinate the expression of genes involved in creating and maintaining the adipocyte phenotype including the insulin-responsive glucose transporter GLUT 4 [2]. Therefore, these transcriptional activators are useful to evaluate adipocyte differentiation pathway.

Recent studies show that obesity may induce systemic oxidative stress; the increase of oxidative species in accumulated fat could be an early instigator of the obesity-associated metabolic syndrome [3]. Oxidative stress plays a critical role in the pathogenesis of several diseases such as cancer, hyperthyroidism, rheumatoid arthritis and diabetes [4–8]. Mitochondria are generally considered as a potential source of cellular reactive oxygen species (ROS) generation [9] but, nonmitochondrial sources of ROS production are known [10–15]. ROS attract current attention not only because of their role in diseases but also as component of intracellular redox signaling cascades [16,17] Treatment of 3T3-L1 with insulin, during differentiation protocol, rapidly generates a burst of hydrogen peroxide, which has a major impact on the early transmission of the insulin receptor signal, suggesting that H_2_O_2_ has a strong impact on insulin signal transduction [18]. Recently, Kim *et al.* demonstrated that exogenous H_2_O_2_ could be able to differentiate preadipocytes to adipocytes increasing C/EBP β and PPAR γ expression [19]. However, others showed that oxidant treatment decreased PPAR γ expression [3]. These different responses to the same oxidant agent shows the complex role that reactive oxygen species (ROS) have in adipocyte differentiation, suggesting that intracellular redox status would play a key role in the modulation transcriptional activator expression. The current study is focused on the contradiction between possible oxidative stress effects on lipid accumulation.

Moreover, high dose of *N*-Acetyl-l-cysteine (NAC) administration to rats, a well known antioxidant, reduces body weight and visceral fat in rats by down regulation of metalothionein expression [20]. Our aim is to evaluate the effect of low dose of NAC on 3T3–L1 differentiation markers expression and cellular lipid accumulation.

## 2. Results and Discussion

### 2.1. NAC Effect on Oxidative Markers

**Figure 1:**
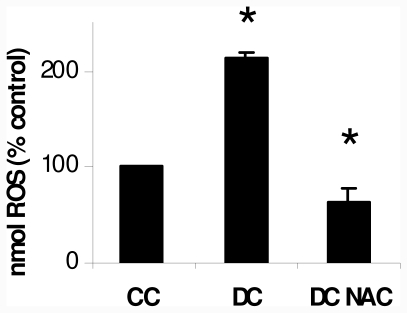
ROS production in 3T3-L1. Intracellular ROS is expressed as nmol ROS (% control), considering Control Cells (CC) as 100. * *p* < 0.01 *vs* control values (CC).

3T3-L1, a preadipocyte cell line is able to differentiate into adipocytes in the presence of MDI (a mixture of 0.5 mM 3-isobutyl-1-methyl xanthine (M), 0.1 μM dexamethasone (D) and, 2 μM insulin (I)), which stimulates triglyceride (Tg) accumulation; the differentiation protocol is developed by culturing the cells with MDI medium in 10% FBS-DMEM for 48 h, and then transferred to fresh 10% FBS-DMEM containing 2 μM insulin. MDI medium addition is considered day 0 of differentiation protocol. At day 10 of differentiation protocol the cells are fully differentiated showed 70–80% cells with refractive lipid droplets observable microscopically, cell cultures showed high concentrations of pro-oxidant species during the cellular differentiation from preadipocytes into adipocytes. In order to evaluate the cellular redox state of adipocytes during differentiation, intracellular reactive oxygen species (ROS) levels were measured using a probe which became fluorescent upon oxidation by ROS; then we quantified its fluorescence. Differentiating cells (DC) at day 10 showed a two-fold higher ROS levels than control cells (CC) (Figure 1). MDI mixture plus 10 μM NAC were added at day 0 of differentiation protocol, NAC was replaced every day during the differentiation protocol, this well known antioxidant reduced ROS levels in differentiating cells at day 10 (DC NAC) to values lower than those obtained in CC.

**Figure 2:**
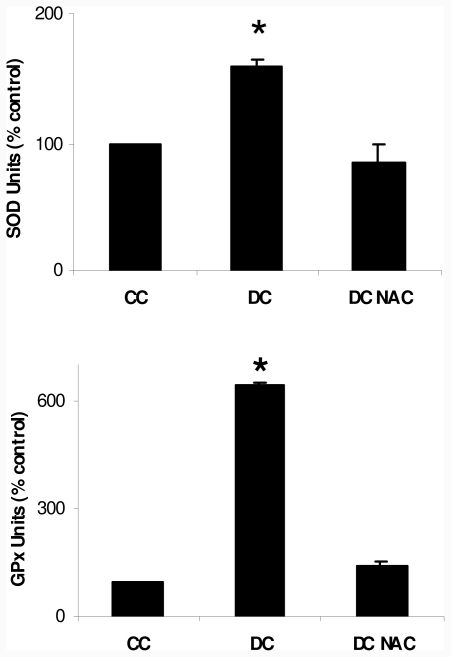
Superoxide dismutase (SOD) activity and glutathione peroxidase (GPX) activity in 3T3-L1. Enzyme activity is expressed as enzyme units (% control), considering Control Cells (CC) as 100%. * p < 0.01 *vs* control values (CC).

Since we observed a pro-oxidant state during differentiation pathway, we determined superoxide dismutase (SOD) and glutathione peroxidase (GPx) activities, as cell responses to possible oxidative stress; we showed that both enzyme activities were significantly higher in DC than in CC at day 10 of differentiation protocol. 10 μM NAC addition reduced both enzymatic activities in DC NAC to values not significantly different from CC; results at day 10 of differentiation protocol are showed in Figure 2.

10 μM NAC was added to cultures during 12 days without any toxic effect, DC and DC NAC cultures showed no significant difference in cell viability, assessed by trypan blue exclusion, during all the differentiation experiment long; we also performed an experiment adding 10 μM NAC to vehicle treated cells (CC), which demonstrated that NAC was not toxic for the cells (data not shown).

Our results showed that a pro-oxidant state was developed in fully differentiated adipocytes (at day 10 of differentiation protocol), in accordance to others authors which had studied this model [21–23]. Nevertheless, Mc Lung *et al.* demonstrated that mice which overexpressed GPx1 (Glutathione peroxidase selenium dependent) developed obesity [24]; since we evaluated ROS and antioxidant enzymes activities at day 10 of differentiation pathway, our results did not invalidate Mc Lung previous results. Moreover, in the absence of an appropriate compensatory response from the endogenous antioxidant network, the system could become overwhelmed; therefore, to avoid ROS increase and their potentially deleterious effects, they are detoxified by several enzymatic systems such as SOD and GPx, as observed in other cell models [25]. Here, enzyme activities increased in 3T3-L1 in fully differentiated adipocytes; NAC, a well known antioxidant, decreased ROS level and, as a consequence, NAC treated cells did not show any antioxidant enzyme activity increase. Therefore we could speculate that NAC did not affect directly antioxidant enzyme activity.

### 2.2. NAC Effect on Triglyceride and GSH Content

At day 10, fully differentiated cells (DC) showed almost six times more triglyceride (Tg) content than in control cells (CC); NAC was added to MDI medium at day 0 of differentiation protocol and replaced every day during 12 days (all differentiation experiment long); then, we determined triglyceride content at day 10 (DCN). We used different concentration of NAC (5 μM, 10 μM, 20 μM, 50 μM and 100 μM) to evaluate the effect of antioxidant. The lower concentration which exerted inhibitory effect on trigliceryde accumulation was 5 μM (0.31 ± 0.05 g Tg/g protein [DCN] *vs* 0.23 ± 0.05 [CC], *p* < 0.01), but 10 μM of NAC was the lower dose that produced the maximum inhibitory effect on triglyceride accumulation since we found in NAC treated cells almost same Tg concentration as obtained in control cells (0.21 ± 0.1 g Tg/g protein [DCN] *vs* 0.23 ± 0.05 [CC], no significant difference was observed); therefore, we used 10 μM NAC for further experiments. NAC did not provoke any effect on control cells, NAC treated cells (CCN) showed similar Tg content to CC (10 μM NAC: 0.20 ± 0.08 Tg/g protein [CCN] *vs* 0.23 ± 0.05 [CC], no significant difference was observed). 5 μM, 10 μM, 20 μM and 50 μM of NAC were used without toxic effects during 12 days, 100 μM of NAC provoked a 30% decrease in the number of viable cells.

**Figure 3:**
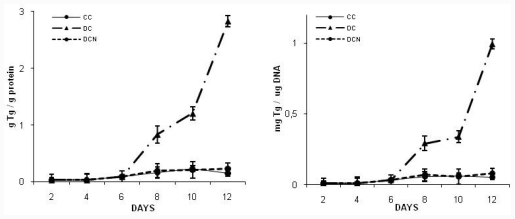
*N*-acetylcysteine (NAC) effect on triglycerides accumulation in 3T3-L1 during differentiation pathway. NAC was added at day 0 and replaced every day, during 12 days (DCN).

A time-course assay to evaluate NAC effect (10 μM NAC) on intracellular Tg levels during cell differentiation pathway showed a consistent significant decrease in Tg content, with a similar kinetic as observed in CC (Figure 3). To exclude errors due to artificial effects of excess fat in differentiated adipocytes, Tg content is reported per protein content and per DNA content. NAC addition to CC did not modify triglyceride content in CC during the experiment.

**Figure 4:**
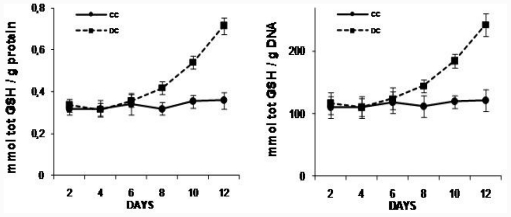
Glutathione (GSH) content in 3T3-L1 during differentiation pathway. NAC was added at day 0 and replaced every day, during 12 days (DCN).

*N*-Acetyl-l-cysteine (NAC), a well known antioxidant, provides cysteine for reduced glutathione (GSH) and promotes its synthesis [26,27]. Since GSH could act as a cellular reductor buffer against pro-oxidant state observed during differentiation preadipocytes pathway, we carried out a time-course experiment to measure the cellular reductor GSH during differentiation protocol. Total GSH level significantly increased at day 8 days of differentiation protocol in differentiating cells (DC) compared to control cells (CC) (Figure 4), coincident with Tg increase in DC. 10 μM NAC addition, from day 0 of differentiation protocol, produced an increase of 25% in intracellular total GSH content in DC from day 1 of differentiation protocol. For instance, at day 10, total GSH was significantly higher in NAC treated DC than in DC (0.53 ± 0.03 mmoles total GSH/g protein [DC] *vs* 0.71 ± 0.04 mmoles total GSH/g protein [DCN], *p* < 0.01). To exclude errors due to artificial effects of excess fat in differentiated adipocytes, total GSH content is reported per protein content and per DNA content.

We observed that intracellular triglyceride content correlates with intracellular total GSH content. Kobayashi also showed that fully differentiated adipocyte had significantly higher GSH content than preadipocytes [28]. GSH is a radical scavenger that directly neutralizes superoxide anion and hydroxyl radicals and it is also an essential cofactor in the inactivation of hydroperoxides by glutathione peroxidase; so, GSH could help as a buffer against ROS allowing cell viability and normal cell function.

Our assays demonstrated that low dose of NAC was able to increase intracellular GSH content since the beginning of differentiation protocol and, it was able to inhibit fat accumulation in 3T3-L1 cells in a dose-dependent manner. Since day 8 of differentiation protocol, Tg content was significantly higher in differentiated cells than control cells; meanwhile, Tg content resulted significantly lower in NAC treated cells than in fully differentiated cells. Also, Tg content in NAC treated cells showed no significant difference to control cells. Similar results were described by Kim *et al.* [20], suggesting that NAC could affect Tg content by metalothionein-II down-regulation. Nevertheless, they showed that doses lower than 2 mM NAC did not affect metalothionein-II expression; therefore we could rule out any possible effect of NAC by this pathway, since we used a 100 times lower dose of NAC. Thus, in this case, we could suggest a NAC effect as ROS scavenging because of GSH increased content.

Since we used low dose of NAC in our experiments, we evaluated a possible reversible effect of this antioxidant. We performed the differentiation protocol as usually, 10 μM NAC was added at day 0 of differentiation protocol and maintained only 24 hours; NAC 24 hours treated cells (DCN*) showed similar Tg content than that observed in DC (day 8: 0.83 ± 0.15 g Tg/g protein [DC] *vs* 0.82 ± 0.18 g Tg/g protein [DCN*], no significant difference was observed; day 10: 1.20 ± 0.10 g Tg/g protein [DC] *vs* 1.16 ± 0.08 g Tg/g protein [DCN*], no significant difference was observed); therefore we observed a reversible effect of the antioxidant on differentiation pathway, since NAC treated cells were able to recover from the treatment. Other authors also showed that NAC should be included early and maintained during differentiation protocol [29], its effect could be in early stage of adipocyte differentiation pathway.

### 2.3. NAC Effect on Adipocyte Differentiation Markers

**Figure 5:**
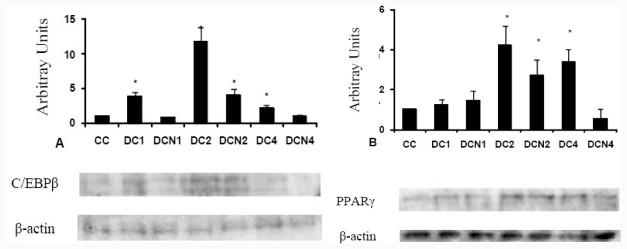
NAC effect on CCAAT/enhancer binding protein beta (C/EBP β) (**A**), and peroxisomal proliferator activated receptor gamma (PPAR γ) (**B**), in 3T3-L1 during differentiation pathway.

It is well demonstrated that treatment of preadipocytes with inducers of differentiation, like MDI medium, produce a transient increase in expression of adipogenic transcriptional factors CCAAT/enhancer binding protein beta (C/EBP β) and peroxisomal proliferator activated receptor gamma (PPAR γ); the cells showed a specific kinetic expression of these transcription factors during differentiation pathway [2]. We could speculate that NAC could interfere with differentiation program of adipocytes; therefore we performed experiments in presence of 10 μM of NAC to evaluate some adipogenic transcription factor expression. We performed a time course analysis for these adipogenic factors in DC (before increase on Tg content was evident). We evaluated adipogenic factors expression from day 0 to day 4 of differentiation protocol in absence and presence of NAC; we added NAC at day 0 and it was replaced every day during all the experiment. C/EBP β (liver-enriched transcriptional activator protein: LAP) showed a similar kinetic expression with/without NAC, but a significant decrease of C/EBP β expression occurred in MDI-NAC treated cells at day 1 (3.90 ± 0.60 AU [DC1] *vs* 0.89 ± 0.10 AU [DCN1], *p* < 0.01), which was supported during the 4 days experiment (Figure 5A). No significant differences for the adipogenic transcription factor PPAR γ expression was observed between DC and MDI-NAC treated cells during first two days of differentiation protocol; however, a significant decrease for PPAR γ was observed at day 4 (3.41 ± 0.60 AU [DC4] *vs* 0.55 ± 0.50 AU [DCN4], *p* < 0.01) (Figure 5(B)).

The values represent fold increase in adipogenic factor expression over the control cells (CC) expression. Representative results from one of three independent western blot experiments with similar results are shown. Results are expressed as arbitrary units; * *p* < 0.01 *vs* control values (CC).

Since NAC significantly affected changes in transcription factors expression; we postulated that NAC inhibited lipid accumulation by affecting at least these two adipogenic transcriptional factors. C/EBP β is expressed immediately after induction of differentiation, and its expression in adipocytes accelerates adipogenesis in response to hormonal inducers [30]. We observed that C/EBP β expression in presence of MDI-NAC resemble differentiated cells pattern; in fact, C/EBP β expression at day 2 in NAC treated cells is higher than that observed in control cells. As previously described, 3T3-L1 cells exhibited the defined pattern for C/EBP β expression during differentiation protocol [31] and, NAC produced a consistent decrease in this adipogenic factor expression during the differentiation with same kinetic of differentiated cells. Therefore, our kinetic experiment demonstrated that NAC affected C/EBP β expression during all differentiation protocol, possibly by increased GSH level which acts as ROS scavenger. We measured ROS level at day 10, but increase in intracellular ROS could be from before that day and linked to the increased expression of C/EBP β at differentiated cells (DC). In accordance with our results, Hallenborg *et al.* demonstrated that high dose NAC produced a decrease on C/EBP β expression after 6 hours of treatment with this antioxidant [29]. On the other hand, Carriere *et al.* [32] showed that cells treatment with drugs used to modulate mitochondrial ROS generation induced a strong increase of CHOP-10 protein content with a slight increase in liver-enriched activating protein (C/EBP β–LAP) during the differentiation pathway. We did not evaluate CHOP-10 expression in our system but, we did a kinetic for C/EBP β (LAP protein), which demonstrated that C/EBP β is lower in MDI-NAC treated cells than that observed in differentiated cells; evidence for C/EBP β DNA binding activity is regulated by a CHOP-10 protein by sequestration of the protein was recently presented [33]. Carriere *et al.* claimed that treatment with these ROS generation inducers decreased adipocytes triglyceride content. Since we did not use any drug to induce ROS during differentiation process and, ROS and triglyceride content increased, as observed by other authors [22], the possible different strategy used by Carriere *et al.* to evaluate adipocyte differentiation process could be responsible for their different results. On the other hand, PPAR γ is a key transcription factor involved in adipocyte differentiation, because of its ability to increase the responsiveness to insulin [34]; its expression in differentiating cells and MDI-NAC treated cells was similar during the first two days of differentiation protocol, only in day 4 we observed a significantly lower PPAR γ expression in MDI-NAC treated cells than in differentiating cells (DC). NAC effect could be explained as a consequence of its antioxidant effect on C/EBP β expression which could affect PPAR γ expression as previously described [35]. These results are in contradiction with those reported by Gou *et al.* [36], but they showed that ROS affected *de novo* triglyceride synthesis instead of adipocyte differentiation pathway, since they added a ROS inducer 6 days after differentiation had started and we added NAC at day 0 of differentiation pathway. Therefore, we could speculate that effect on endogenous adipogenic factors was largely subevaluated by Gou *et al.*, because PPAR γ increase expression is observed at day 4, before any accumulation of triglycerides occurred; meanwhile we observed an increase on ROS level during differentiation and NAC deeply affected adipogenic factor expression during adipocyte differentiation. Recently, Kim *et al.* showed that replacement of insulin by H_2_O_2,_ at day 0 of differentiation protocol, could increase C/EBP β and PPAR γ expression in 3T3-L1, suggesting that ROS, in the beginning of differentiation pathway, could facilitate pre-adipocyte differentitation [19]. We observed that intracellular triglyceride content correlates with intracellular GSH content, and GSH levels increased after adipogenic transcriptional factors expression took place. Nevertheless, 10 μM NAC addition at day 0 produced an increase in intracellular GSH content and a decrease in adipocytes differentiation markers expression, suggesting that GSH level should prevent C/EBP β expression increase and therefore PPAR γ increase.

**Figure 6:**
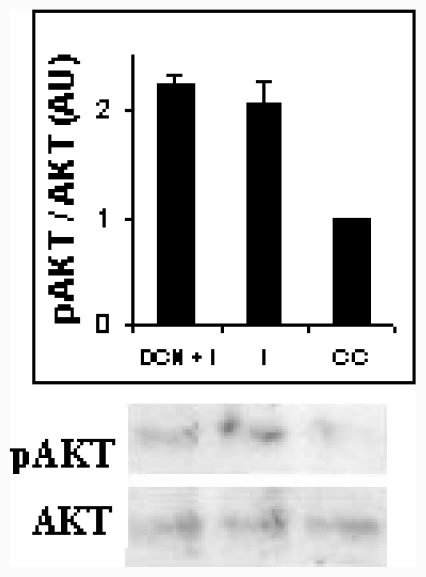
NAC effect on AKT phosphorylation in 3T3-L1. Representative results from one of three independent western blot experiments with similar results are shown. Results are expressed as arbitrary units.

IGF–1 and Insulin receptors are structurally similar and mostly have common signaling cascades, both insulin and IGF-1 stimulated ROS production, and some authors suggested that IGF-1 exerts its effect at least in part via ROS [37]. We could presume that NAC could block initial step of adipocyte differentiation via inhibiting insulin by disturbing functional structure of insulin molecule. Therefore, we assayed NAC effect on a primary target of insulin cascade signaling such as AKT. We performed an experiment to evaluate AKT phosphorylation; we did not observe any significant difference between Insulin with NAC treated cells (DCN+I) and Insulin treated cells (I) (Figure 6); therefore, insulin was able to provoke AKT phosphorylation in NAC presence. Our results suggested that functional structure of insulin molecule was not disturbed by NAC and insulin could act by this pathway. In our case, we could explain NAC effect by its capacity as GSH precursor. Indeed, other authors demonstrated that GSH could decrease AKT phosphorylation when it was added to differentiated adipocytes [28].

We observed an effective and reversible effect of NAC in this system, suggesting that GSH content could be primordial to maintain C/EBP β expression inhibition and triglyceride accumulation inhibition. Indeed, mice which consumed high-fat diet with NAC had significantly lower body weight than high-fat diet water group [38]. Therefore, we could speculate that a minimum level of ROS is necessary during differentiation pathway; recently, 4-hydroxynonenal (HNE), a product of lipid peroxidation has been proposed by several authors as an intracellular signaling mediator, rather than a toxic product of lipid peroxidation [39,40]. HNE promotes a great increase in the expression of PPAR γ in human leukemic cells [41,42]. Ristow *et al.* have shown that transient increase of oxidation stress by ROS induced PPAR γ expression in human muscle as well as a secondarily increase expression of ROS detoxifying enzymes including SOD 1, SOD 2 and GPx1, suggesting that ROS mediated PPAR γ increase as our results. Another kind of antioxidants, which do not impact the amount of GSH, such as Vitamin E and C provoked decreased of PPAR γ mRNA [43]. Some authors reported that flavonoids and phenolic acids suppress adipogenesis in 3T3-L; o-coumaric acid, rutin and luteolin had the highest inhibition on triglyceride accumulation and, also inhibited the expression of PPAR γ during cellular differentiation [44,45] Between antioxidants, which do not affect GSH content, one of the most interesting of them is tocotrienol, which suppress adipocyte differentiation and AKT phosphorylation in 3T3-L1 preadipocytes [46].

## 3. Materials and Methods

### 3.1. Cell Cultures and Drugs

Swiss 3T3-L1 (mouse preadipocytes) was obtained from the American Type Culture Collection (Rockville, MD, USA). Cells were cultured in Dulbeco Modified Eagle Medium with 25 mM glucose (DMEM) supplemented with 10% fetal bovine serum, pH 7.4, at 37 °C, under 5% CO_2_. *N*-Acetyl-l-Cysteine, fluorescent probe CM-H2DCFDA and all other reagents (except when specified) were purchased from Sigma-Aldrich Co, St. Louis, MO, USA.

### 3.2. Differentiation of 3T3-L1 Preadipocytes into Adipocytes

3T3-L1 cells (a subclone derived from the Swiss 3T3 parental line) differentiation can be achieved in one week by culturing the cells with MDI medium (a mixture of 0.5 mM 3-isobutyl-1-methyl xanthine (M), 0.1 μM dexamethasone (D) and, 2 μM insulin (I)), in 10% FBS-DMEM for 48 h, and then transferred to fresh 10% FBS-DMEM containing 2 μM insulin. MDI addition is considered day 0 of differentiation protocol; after this treatment, 70–80% cells dramatically increased their triglyceride content in 10 days, generating refractive droplets easily observable microscopically by conventional or dark-field microscopy, or by Oil Red O staining [47]. We considered MDI treated cells which undergoing differentiation pathway by the differentiation protocol as differentiating cells (DC) and, vehicle treated cells as control cells (CC). In indicated plates 5 or 10 μM *N*-acetylcysteine (NAC) was added at day 0 of differentiation protocol, in this case we added NAC plus MDI medium (DCN).

At indicated times, according with the experiment, the cells were washed once with 0.01 M phosphate buffered saline at room temperature, and 0.5 mL water was added to each culture. Cells were scraped and frozen until chemical determinations were performed. Results are the average of four different experiments (mean ± SD). In the case of western blot analysis cells were cells were lysed with a buffer containing 1% SDS in 60 mM Tris-HCl, boiled for 10 minutes and, centrifuged at 15,000 rpm at 4 °C for 10 minutes and used to perform the experiment. Results are the average of three independent experiments (means ± SD).

### 3.3. Determination of Triglyceride

Triglyceride accumulation was determined using TG color GPO/PAP AA kit (Wiener Laboratory, Rosario, Argentina). Results are the average of four different experiments (means ± SD). Triglyceride content is expressed as g Tg/g protein or mg Tg/ug DNA.

### 3.4. Glutathione Determination

Total glutathione (GSH plus GSSG) concentration was determined by a DTNB-GSSG reductase recycling procedure. GSH is oxidized by 5,5′-dithiobis-2-nitrobenzoic acid (DTNB) to give GSSG with stoichiometric formation of 5-thio-2-nitrobenzoic acid (TNB). GSSG is subsequently reduced to GSH by the action of the highly specific glutathione reductase and NADPH, releasing a second TNB molecule and recycling the GSH. Any oxidized GSH (GSSG) initially present in the reaction mixture is rapidly reduced to GSH. The rate of TNB formation is followed at 405nm and is proportional to the sum of GSH and GSSG present (total glutathione). The amount of GSH is determined from a standard curve in which GSH equivalents present is plotted against the rate of change of absorbance at 405 nm [48]. Results are the average of four different experiments (means ± SD).

### 3.5. Determination of Protein and DNA

Proteins were determined by Bradford method using crystalline bovine serum albumin as standard [49]. DNA content was determined at 260 nm [50].

### 3.6. Determination of Superoxide Dismutase

Enzymatic activity was measured in cell extracts. Superoxide dismutase (SOD, EC 1.15.1.1) was determined using RANDOX kit, which follows a method based on that originally described by Mc Cord and Fridovich [51]. This method involves the use of xanthine and xanthine oxidase (XO) system to generate superoxide radicals (O_2_^·−^), which react with 2-(4-iodophenyl)-3-(4-nitrophenol)-5-phenyltetrazolium chloride (INPPT) to form red formazan dye (a). SOD present in the sample compete with INPPT for superoxide radicals and inhibits the production of the formazan dye (b). SOD was determined measuring the degree of inhibition of formazan dye formation exerted by cell extracts [52]. Results are the average of four different experiments (means ± SD).

### 3.7. Determination of Glutathione Peroxidase

Enzymatic activity was measured in cell extracts. Glutathione peroxidase selenium dependent (EC 1.11.1.9) was determined using RANDOX kit by a method developed by Paglia and Valentine [53]. This method is based on enzyme reaction in which glutathione peroxidase (GPx) catalyzes glutathione (GSH) oxidation (a) by cumene hydroperoxide (ROOH) and the product oxidized glutathione (GSSG) is used to drive the oxidation of NADPH + H^+^ catalyzed by glutathione reductase, with concurrent reduction of GSSG (b). The concentration of GPx is assessed from the decrease in absorption at 340 nm due to oxidation of NADPH to NADP^+^. Results are the average of four different experiments (means ± SD).

### 3.8. Measurement of Intracellular ROS Generation

Cells were washed with 0.01 M phosphate buffered saline at room temperature and then incubated in the dark with a cell-permeable non-fluorescent probe CM-H2DCFDA (10 μM in Krebs-Ringer bicarbonate buffer) for 30 min at 37 °C. The probe is de-esterified intracellularly and turns to highly fluorescent 2′,7′-dichlorofluorescin upon oxidation. Fluorescence emission spectra (between 515 and 540 nm) was monitored in a QuantaMaster Model QM-1 PTI spectrofluorometer, and corrected for detector sensitivity and monochromator blaze angle by the software of the equipment, with an excitation wavelength at 505 nm. The fluorophore incorporated in cell cultures was 2′,7′-dichlorodihydrofluorescin diacetate, a probe that becomes fluorescent under oxidation and emits green fluorescence. ROS production was determined from a H_2_O_2_ standard curve (0.1–5 μM). The fluorescence intensities of H_2_O_2_ solutions were plotted *vs* H_2_O_2_ nmoles, and it was observed that fluorescence intensity increased at higher oxidant concentrations until the system is saturated. Thus, the best fittings for a linear regression were obtained with inverted plots. All the experiments were measured at room temperature. Results of four independent experiments were normalized by protein content (means ± SD).

### 3.9. Western Blot Analysis

3T3-L1 cells were lysed with a lysis buffer (1% SDS, 60 mM Tris-HCl), boiled for 10 minutes and, centrifuged at 15,000 rpm at 4 °C for 10 minutes. Proteins (10 μg of different samples) were then separated on 15% SDS-polyacrylamide gel and transferred to nitrocellulose membranes (Amersham, GE Healthcare), which were soaked in blocking buffer (BSA 0.1%, Tween 0.4% and EDTA 1mM in 0.01 M phosphate buffered saline) for 1 hour, and then incubated overnight with a mouse monoclonal antibody against PPAR γ (PPAR γ (E-8) purchased from Santa Cruz Biotechnology), with a rabbit monoclonal antibody against C/EBP β which recognized LAP protein (C/EBP β (C-19) purchased from Santa Cruz Biotechnoloy), with antibodies directed against Akt and phosphoSer^473^-Akt (purchased from Cell Signaling Technology) or rabbit anti-β actin antibody (anti-β actin 20–33 purchased from SIGMA) at 4 °C. Horseradish peroxidase-conjugated secondary antibodies (anti-mouse Ig G Santa Cruz Biotechnology or anti-rabbit Ig G SIGMA) and an enhanced chemiluminescence (ECL) substrate kit (Amersham ECL Plus Western Blotting Detection System, GE Healthcare) were used for detection of specific protein. Adipogenic factors expression (PPAR γ and C/EBP β) was normalized by β-actin expression in each sample.

Samples were obtained at day 1, day 2 and day 4 of differentiation protocol (DC1 and DCN1: day one of differentiation protocol, DC2 and DCN2: day two of differentiation protocol, DC4 and DCN4: day four of differentiation protocol). In all the cases, expression was normalized by β-actin protein expression. Results of each adipogenic factor are the average of three different experiments (means ± SD); they are expressed as arbitrary units.

### 3.10. Akt Assays

Akt assays were performed according to the manufacturer’s protocol, using 40 μg of 3T3-L1 lysate protein in buffering containing 60 mM Tris (pH 7.4) and 100 mM NaCl for western blot analysis. Briefly, the experiment was carried out by culturing the cells were in medium containing 1% FBS overnight to reduce basal levels of phosphorylation; then 2 μM insulin or 2 μM insulin with 10 μM NAC was added to the cells. After the treatment, the cells were lysed with a lysis buffer (1% SDS, 60 mM Tris-HCl, 100 mM NaCl), boiled for 10 minutes and, centrifuged at 15,000 rpm at 4 °C for 10 minutes, and kept in ice until western blot analysis was performed. We used antibodies directed against Akt or phosphoSer^473^-Akt; they are expressed as arbitrary units, the results of each assay are the average of three independent experiments (means ± SD).

### 3.11. Statistics

Results are expressed as means ± SD. Statistical analysis was performed by one-way analysis of variance followed by post-anova analysis [54].

## 4. Conclusions

We demonstrated that NAC addition inhibited triglyceride accumulation in adipocytes, by preventing the increase of ROS and antioxidant enzyme activities during differentiation. NAC increased intracellular GSH content and, as a consequence, the antioxidant reduced the differentiation markers expression. Therefore, in this system, NAC seems to exert a beneficial effect on the prevention of metabolic changes induced in adipocytes during obesity development. NAC is currently used pharmacologically as a mucolytic agent in a variety of respiratory illnesses; however, now it appears to also have beneficial effects in conditions characterized by oxidative stress, such as heart disease, cigarette smoking and cancer. Here, we evaluated NAC inhibitory effect on trigliceryde accumulation, which is relevant not only for gaining insight into pathogenesis of metabolic disorders, but also for identifying pathways which might be appropriate targets for pharmacological interventions. Diets with antioxidants, such as NAC, could have relevance in regulation of antioxidant system and free radical production.

## Abbreviations

GSH: Glutathione
NAC: *N*-acetylcysteine
C/EBP β: CCAAT/enhancer binding protein beta
PPAR γ: peroxisomal proliferator activated receptor gamma
